# What Is the Assembly Pathway of a Conjugated Polymer From Solution to Thin Films?

**DOI:** 10.3389/fchem.2020.583521

**Published:** 2020-12-22

**Authors:** Zhuang Xu, Kyung Sun Park, Ying Diao

**Affiliations:** ^1^Department of Chemistry, University of Illinois Urbana-Champaign, Urbana, IL, United States; ^2^Department of Chemical and Biomolecular Engineering, University of Illinois Urbana-Champaign, Urbana, IL, United States

**Keywords:** organic electronics, crystallization, liquid crystal, pre-aggregation, assembly pathway, molecular assembly, conjugated polymers

## Abstract

The hierarchical assembly of conjugated polymers has gained much attention due to its critical role in determining optical/electrical/mechanical properties. The hierarchical morphology encompasses molecular-scale intramolecular conformation (torsion angle, chain folds) and intermolecular ordering (π–π stacking), mesoscale domain size, orientation and connectivity, and macroscale alignment and (para)crystallinity. Such complex morphology in the solid state is fully determined by the polymer assembly pathway in the solution state, which, in turn, is sensitively modulated by molecular structure and processing conditions. However, molecular pictures of polymer assembly pathways remain elusive due to the lack of detailed structural characterizations in the solution state and the lack of understanding on how various factors impact the assembly pathways. In this mini-review, we present possible assembly pathways of conjugated polymers and their characteristics across length scales. Recent advances in understanding and controlling of assembly pathways are highlighted. We also discuss the current gap in our knowledge of assembly pathways, with future perspectives on research needed on this topic.

## Introduction

**Figure 1:**
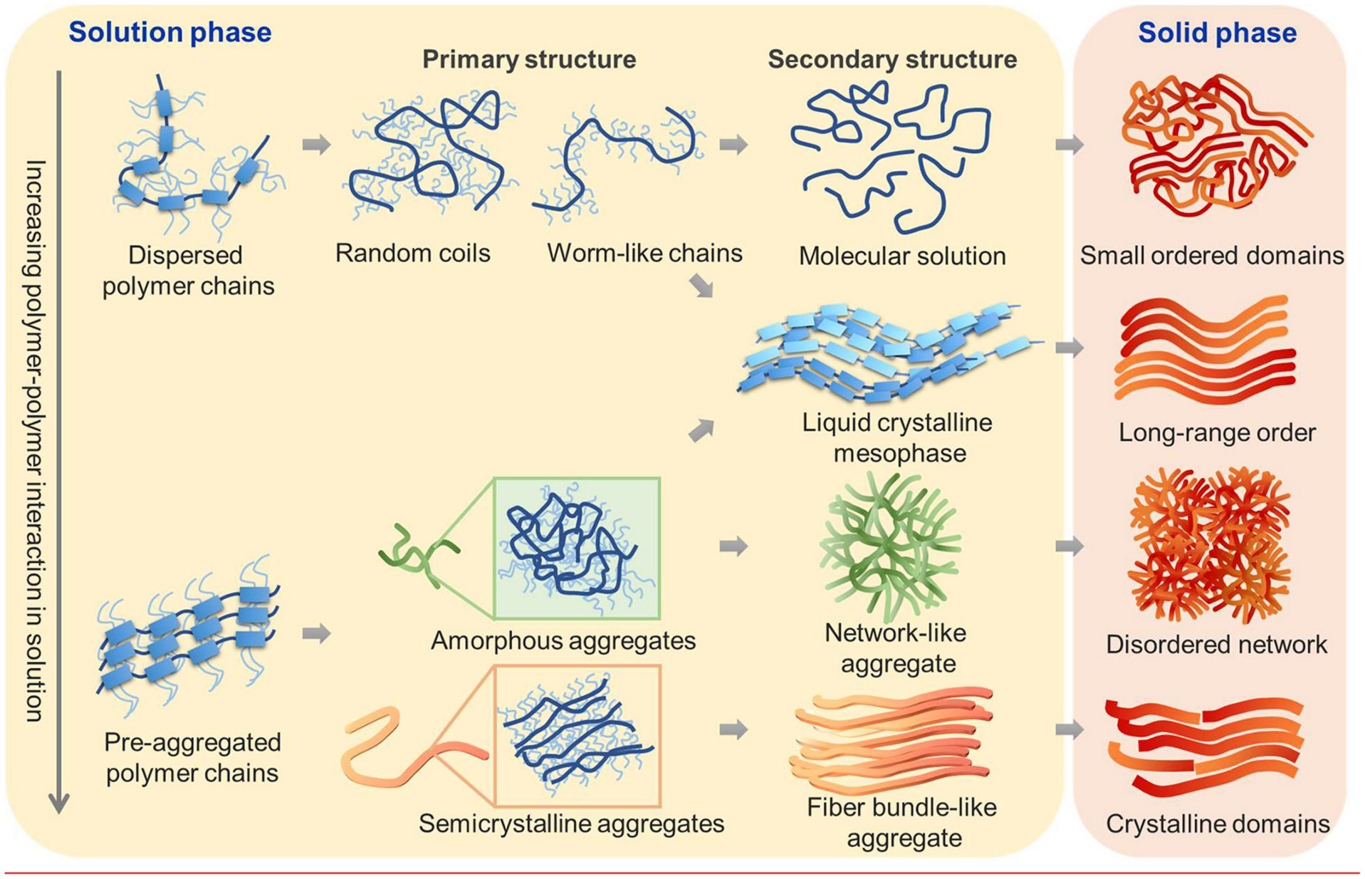
Schematic illustration of possible molecular assembly pathways of conjugated polymers from solution to thin films. Relative strengths of polymer–polymer and polymer–solvent interactions determine the initial state of polymers in solution. Fully dispersed polymer chains of various conformations can directly crystallize into semicrystalline thin films featuring small-ordered domains or can first assemble into a liquid crystalline mesophase which results in films with long-range order. Alternatively, polymer chains can pre-aggregate in the solution state to form disordered or semicrystalline aggregates as the primary assembled structure. These primary aggregates can further assemble into secondary aggregates of various types, including but not limited to liquid crystalline mesophase, network-like or fiber-bundle-like aggregates, which then determine the morphology of thin films.

Organic semiconductors have undergone remarkable development as promising materials for next-generation electronics due to their mechanical flexibility, stretchability, and self-healing properties (Someya et al., 2016), ability to interface and communicate with biological systems (Wang et al., 2018), and compatibility with high-throughput, low-cost manufacturing (Gu et al., 2018). In conjugated polymers, the electronic performance sensitively depends on the morphology across multiple length scales such as polymer conformation, packing, crystallinity, alignment, and domain connectivity (Noriega et al., 2013; Diao et al., 2014; Venkateshvaran et al., 2014; Himmelberger and Salleo, 2015; Patel and Diao, 2018; Gu and Loo, 2019). Controlling assembly pathways of conjugated polymers can offer large modulation of their multiscale morphology for enhancing ultimate device performance. Here we discuss three main types of conjugated polymer assembly pathways from solution to solid films: (i) direct crystallization/aggregation from dispersed polymer chains, (ii) pre-aggregation-mediated assembly, and (iii) liquid crystal (LC)-mediated assembly (Figure 1).

The first assembly pathway involves fully solubilized polymer chains that directly crystallize from solution without traversing intermediate aggregated states. It is usually found when the polymer–solvent interactions dominate over polymer–polymer interactions in a good solvent and/or at high temperature. Depending on the intrinsic molecular structures and polymer–solvent interactions, a variety of polymer chain conformation is possible from random coil (Ikai et al., 2016), collapsed coil (Traiphol et al., 2007), ribbon-like chains (Root et al., 2017), and worm-like cylinder (Root et al., 2017) to rigid rods (Cotts et al., 1996) which crucially impact chain conformation, packing, and charge transport in the solid state. In conjugated polymers, the main intermolecular forces including π–π interactions between the backbones and dispersion forces among the alkyl side chains often result in the formation of pre-aggregates and/or LC mesophase in solutions. When the intermolecular interaction of polymers is strong, pre-aggregates are readily developed in the solution state even at low concentrations. Donor–acceptor copolymers, for instance, may be prone to pre-aggregation because intramolecular charge transfer from donor to acceptor moieties gives rise to more planarized backbone as well as strong intermolecular interaction between neighboring molecules. The internal structure of the pre-aggregates can be amorphous or semi-crystalline, depending on the molecular structures and the type of interactions (Figure 1). Amorphous pre-aggregates can result from weak π–π interactions or side chain association/interdigitation, whereas semi-crystalline pre-aggregates arise from strong the π–π stacking of the backbone and/or side chain crystallization. In both cases, the pre-aggregates usually take the form of polymer fibers with a diameter in the nanoscopic scale. Such primary pre-aggregates can further associate to form secondary aggregates in solution, usually of microns in size or above. The secondary aggregate may adopt a fiber bundle-like morphology which further grows into highly crystalline thin films or a network-like aggregate which evolves into a disordered fiber network morphology in thin films. Besides the direct crystallization and the pre-aggregation-mediated pathways, LC mesophase-mediated pathways are often observed, which can result in long-range order in the solid state. While the thermotropic LCs of conjugated polymers have long been studied (Sirringhaus et al., 2000; McCulloch et al., 2006; Lee et al., 2011), lyotropic LCs are gaining interests in recent years due to its direct relevance to solution processing that involves evaporative assembly. Furthermore, lyotropic LC mesophases are conducive to shear alignment during solution processing for enhanced charge transport. Lyotropic LC mesophases can form either from dispersed polymer chains or from pre-aggregated polymer nanofibers when their volume fraction surpasses a threshold (Zhang S. et al., 2010; Bilger et al., 2017). In other words, the mesophase can be either “molecular” or “colloidal” LCs. In either case, the anisotropic shape of the constitutive molecule/particle and/or their amphiphilic interactions can play important roles in inducing mesophase formation (Hendrikx et al., 1983; Alexandridis et al., 1998).

Currently, there is a gap in our knowledge regarding what determines the assembly pathways of semiconducting polymers from solution to thin films and how the assembly pathways can be tuned by design. While recent studies surmised the important role of pre-aggregation and LC mesophase in crystallization and alignment of conjugated polymers, several aspects regarding the nature of intermediate states and their multiscale structures remain unclear. For example, poly{[*N,N*′-bis(2-octyldo-decyl)-1,4,5,8-naphthalenediimide-2,6-diyl]-*alt*-5,5′-(2,2′-bithiophene)} [P(NDI2OD-T2)] has been known to form pre-aggregates easily in common organic solvents (Steyrleuthner et al., 2012; Nahid et al., 2018). It has been claimed that pre-aggregation is beneficial to inducing a local ordered structure, giving rise to better transistor performance (Luzio et al., 2013; Nahid et al., 2018). On the other hand, it was shown that P(NDI2OD-T2) can form a LC mesophase which is possibly developed from the pre-aggregation (Trefz et al., 2019). However, it is still unknown whether pre-aggregates are prerequisite to develop the LC mesophase and what the specific structures of pre-aggregates and LC mesophases are. To fill this knowledge gap, it is critically important to gain a fundamental understanding of assembly pathways, as they can determine all the morphology features in thin films and thus solid-state properties. However, previous work has frequently overlooked the journey of the polymers from solution to thin film and focused only on the final morphology and property of thin films. We believe that “the journey is at least as important as the destination” and therefore choose to highlight several insightful studies on three distinct assembly pathways and various factors that impact assembly pathways in this minireview.

## Conjugated Polymer Assembly From Dispersed Polymer Chains

**Figure 2:**
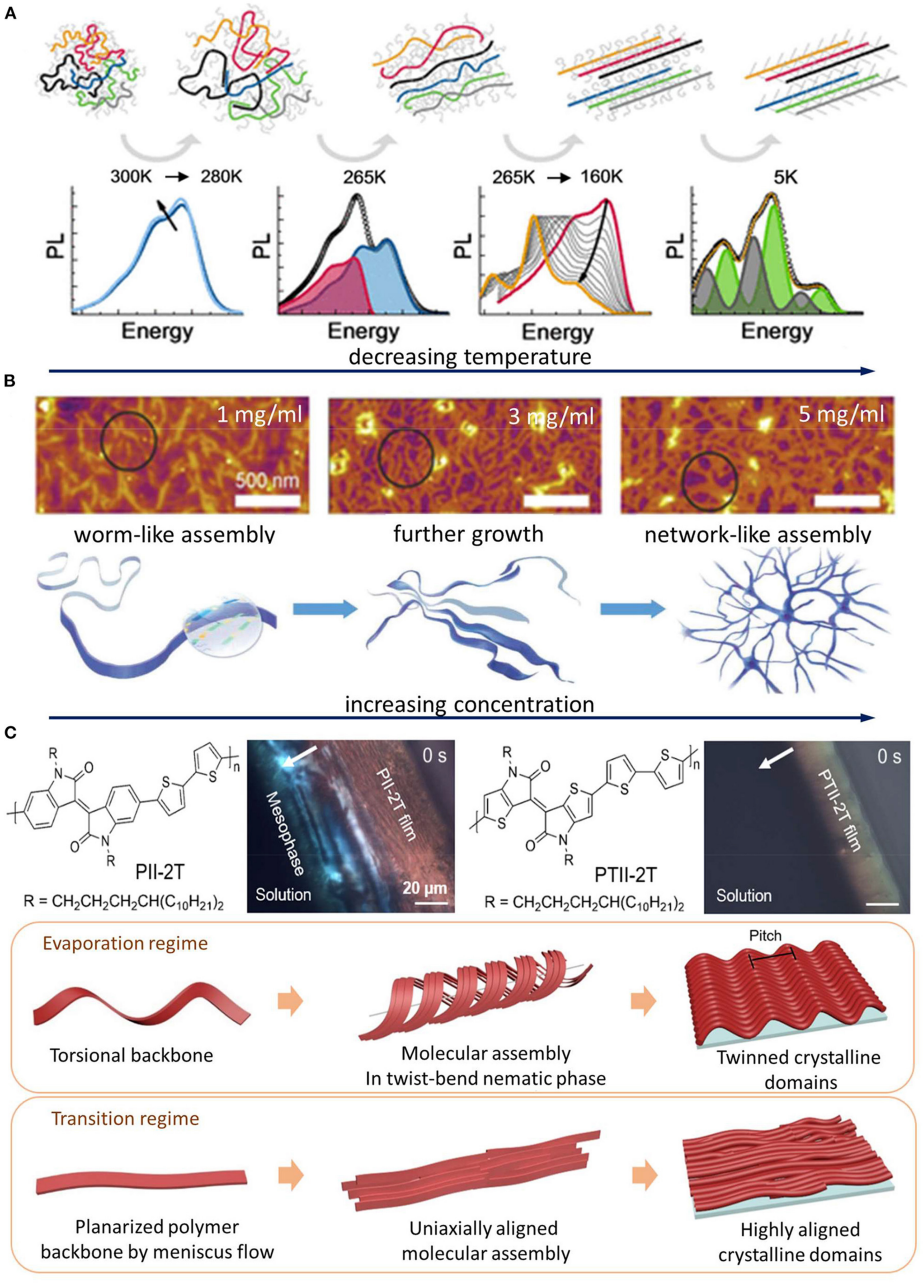
Examples of direct crystallization, pre-aggregation, and liquid crystalline (LC)-mediated assembly pathways. **(A)** Direct crystallization pathway: elementary steps of temperature-induced direct crystallization from dispersed polymer chains and corresponding photoluminescence spectra for P3HT solution. The image was adapted from Panzer et al. (2017) with permission from the American Chemical Society. **(B)** Pre-aggregation pathway: atomic force microscopy images of freeze-dried benzodifurandione-based oligo(*p*-phenylene vinylene) polymers from chloroform solutions at various concentrations and corresponding schematic showing the morphology development from 1D pre-aggregates to 2D network like structures. The image was adapted from Yao et al. (2019) with permission of Wiley. **(C)** LC mesophase pathway: chemical structures of PII-2T and its planar counterpart PTII-2T and their CPOM image comparison during solution-to-solid-state phase transition in a receding meniscus. PII-2T shows a strong birefringence near the contact line, indicating LC mesophase formation. In contrast, such mesophase is missing for PTII-2T. The schematic beneath illustrates that the appearance of LC mesophase of PII-2T is modulated by printing flow–torsional polymer chains assemble in a helical fashion to form a *N*_TB_ mesophase in the evaporation regime. In the transition regime, the higher strain stretches out the twisted chains to eliminate the *N*_TB_ mesophase, resulting in uniaxially aligned morphology. The image was adapted from Park et al. (2019), with permission from AAAS.

When dissolved in a good solvent mutual to both the conjugated backbone and the side chain, conjugated polymers are present in the form of dispersed polymer chains (McCulloch et al., 2013; Newbloom et al., 2015; Santos et al., 2016). Early works on poly(3-hexylthiophene) (P3HT) have shown that the aggregation and crystallization of dispersed polymer chains can be commonly induced by lowering T (Samitsu et al., 2008; Koppe et al., 2009), decreasing the solvent quality (Park et al., 2009; Sun et al., 2011; Keum et al., 2013; Baghgar et al., 2014). An elegant work by Panzer et al. has probed the elementary steps of conjugated polymer crystallization from fully dispersed polymer chains using *in situ* UV–*Vis* and photoluminescence spectroscopy (Panzer et al., 2017). In this work, crystallization was initiated by cooling an enclosed vial of dilute solution, distinct from evaporative crystallization during solution coating/printing. The work unveiled a general assembly pathway for three different types of compounds—homopolymers, donor–acceptor-type polymers, and low molecular weight compounds. The shared pathway features chain expansion before collapsing into a highly ordered dense state. Figure 2A illustrates a specific example for P3HT where the coiled chains first swell and undergo planarization to form disordered aggregate. Further lowering the temperature leads to backbone planarization and crystallization from the disordered aggregate. Eventually, additional side chain crystallization takes place (separately from backbone crystallization) to form crystalline aggregates. The pronounced change in temperature-dependent UV–*Vis* and photoluminescence spectra informs each distinct molecular conformation and aggregation state. It should be noted that, in this case, the assembly into aggregates is induced by decreasing the solvent quality without changing the solution's concentration. For common solution processing techniques for conjugated polymers, however, the assembly process is likely different as it is driven by solvent evaporation wherein the solution traverses the entire concentration range.

## Pre-Aggregation-Mediated Assembly Pathway

Conjugated polymers are pre-aggregated in solution when polymer–polymer interactions in solution overcome the polymer–solvent interactions. A pioneering work by Nguyen *et al*. showed that pre-aggregates of MEH–PPV can be preserved through the assembly process to determine the film morphology and optoelectronic properties (Nguyen et al., 2000). Because the structural features within the pre-aggregates carry over to the solid state, it is critical to understand the multi-level structures in the pre-aggregates. In recent years, there has been considerable interest in studying the pre-aggregation of conjugated polymers as it often leads to desired morphology such as polymer alignment and enhanced electrical properties in films (Park et al., 2011; Aiyar et al., 2013; Kleinhenz et al., 2015; Wang et al., 2015, 2017; Nahid et al., 2018; Kim et al., 2019; Xie et al., 2019; Zhang et al., 2019; Wu et al., 2020). Besides alignment, pre-aggregation can also modulate other aspects of thin film morphology such as dimensionality and out-of-plane molecular orientation. For instance, a recent study has shown dip-coating of wafer-scale polymer monolayer transistors by fine-tuning the degree of pre-aggregation using various solvents and solution concentrations (Yao et al., 2019). Figure 2B shows how benzodifurandione-based oligo(*p*-phenylene vinylene) (BDOPV) polymers are self-assembled in chloroform solution at different solution concentrations. One-dimensional (1D) worm-like aggregates initially formed in solution *via* a strong π–π interaction as revealed by small-angle neutron scattering (SANS). The further growth of those 1D aggregates driven by solvent evaporation led to the formation of two-dimensional (2D)-like networks. This solution-state assembly enabled highly uniform 2D monolayers under the optimal dip-coating condition. Furthermore, controlling pre-aggregation enables tuning the molecular orientation of conjugated polymers (Li et al., 2016). A different degree of aggregation for difluorobenzothiadiazole-based polymers resulted from chloroform (CF) and 1,2,4-trichlorobenzene (TCB). The CF system produced highly aggregated fibrillar features in drop-cast films, whereas gradually adding TCB into the CF solution lowers the extent of pre-aggregation in solution as evidenced by the solution UV–*Vis* spectra and atomic force microscopy images of thin films drop-cast from mixed solvents. Interestingly, the films produced from solutions in CF and TCB exhibited fully edge-on and face-on molecular arrangement, respectively. The authors explained this phenomenon by how the different type of pre-aggregation interacts to the substrate surface, namely, a competition between polymer–polymer and polymer–substrate surface interaction. They believe that relatively isolated chains in TCB adopt a lying-flat orientation to maximize their interactions with substrates (silicon wafers with 300-nm-thick thermally grown SiO_2_ functionalized by hexamethyldisilazane), resulting in a face-on orientation. In CF, however, they speculate that the intermolecular π–π interaction is strong enough to compensate the energy required for the molecular reorientation of polymers to “stand up” on the substrate; thus, an edge-on orientation is expected (Al-Mahboob et al., 2013). Due to the fact that the edge-on orientation led to a π-stacking direction which is parallel to the conduction channel in most studied organic field-effect transistors (Sirringhaus et al., 1999), the charge carrier mobility was enhanced by more than two orders of magnitude in CF compared to that in TCB. This study presents a case study in which the desired molecular orientation can be obtained by tuning the pre-aggregation pathways.

How can the extent of polymer pre-aggregation in solution be controlled? Parameters such as the choice of solvent, dissolution temperature, and solution aging time are important factors used to tune pre-aggregation-mediated assembly pathways. Zheng et al. have reported that the pre-aggregated structure of BDOPV-based polymers can be tuned by the choice of solvent (Zheng et al., 2017). By solution SANS measurements and electron microscopy imaging of freeze-dried samples from solution states, the authors inferred 1D rod-like and 2D lamellar structures in ODCB and toluene solutions, respectively. The intermediate state between those two structures was found in the mixed solvents. Notably, the devices made from the mixed solvent exhibited the highest electron mobility among those prepared from the neat solvent. The authors attributed this result to the favorable formation of tie chains between the aggregates induced in the mixed system. They claimed that the addition of toluene, which is a poor solvent, into ODCB solution promotes secondary polymer aggregation without changing the primary aggregation structure. Only in mixed solvent was the connectivity between the aggregates promoted, leading to a highly ordered solid-state packing and thus a better charge transport property. This work has shown that a delicate control over the structure of pre-aggregates is achievable through proper solvent selection, which can be critical for optimizing device performance. Very recently, a simple strategy has been developed to tune pre-aggregation-mediated assembly pathway *via* polymer dissolution temperature (*T*_dis_) at which solutions were prepared (Li et al., 2020). It is suggested that stronger aggregation with extended π–π interactions can be induced when decreasing *T*_dis_, resulting in a significant increase in the width of the fibrils. Importantly, the charge transport property of the resulting monolayer was highly enhanced due to the improved molecular ordering. They proposed that a low *T*_dis_ boosts the growth rate of aggregates by almost three-fold. The results have demonstrated that *T*_dis_ is an important factor for modulating the size of the pre-aggregates. Besides that, a study conducted by Chu et al. has shown that the pre-aggregation level of P3HT in solution increases with aging time (Chu et al., 2016). Pre-aggregation bonded polymer chains together by π–π interactions to form nanofibers in solution, which resulted in reduced tortuosity and entanglement during blade-coating. As a result, the alignment of polymer chains in solid state was greatly improved with increased polymer pre-aggregation, which can be precisely controlled by the solution's aging time prior to the solidification process.

## Liquid Crystal-Mediated Assembly Pathway

The LC-mediated assembly pathway of conjugated polymers has been extensively utilized for achieving a highly aligned thin film morphology. Earlier works on thermotropic liquid crystalline-conjugated polymers such as poly(2,5-bis(3-alkylthiophen-2-yl)thieno[3,2-*b*]thiophene) and poly-9,9′dioctyl-fluorene-co-bithiophene have shown that thermal annealing-induced LC mesophase transformation can enhance the molecular order and charge transport property in thin films (Sirringhaus et al., 2000; McCulloch et al., 2006; Delongchamp et al., 2008, 2009; Zhang X. et al., 2010; Lee et al., 2011; Snyder et al., 2015). Several theoretical studies by Xie et al. have also investigated the nematic ordering of conjugated polymers (Zhang et al., 2015, 2016; Xie et al., 2018) and its impact on charge transport properties (Zhang et al., 2018). Lyotropic conjugated polymers have recently been investigated by a growing body of literature due to their direct relevance to solution processing (Zhang S. et al., 2010; Bilger et al., 2017; Yang et al., 2018; Trefz et al., 2019). Thus, there has been increasing interest in understanding/controlling LC-mediated assembly pathways and synthetic approaches to facilitate the formation of lyotropic LC (Park et al., 2011, 2019; Kim et al., 2013; Kleinhenz et al., 2015; Bridges et al., 2016, 2017; Chung et al., 2019). Bridges et al. have developed a strategy to induce LC mesophases by changing the length and bulkiness of the alkyl side chains on PCDTPT (Bridges et al., 2016). When the alkyl chains are longer and bulkier, the amphiphilic polymers become soluble in hexane, which has selective affinity toward the side chains. The aromatic backbones are poorly soluble in hexane and therefore extend and stack *via* π–π interactions to reduce solvophobic interactions. This results in a lyotropic nematic LC exhibiting birefringence under a cross-polarized optical microscope (CPOM). Interestingly, by changing the selective solvent (hexane) to a mutual solvent (chloroform or toluene) that solubilizes both the backbone and the side chains, no lyotropic LC phase, but an isotropic solution, is observed at the same solution concentration for polymers with long and bulky side chains. By further decreasing the solubility in mutual solvent (shorter, less bulky side chain), pre-aggregates form in place of isotropic solutions. This is an elegant example showing how to access different assembly pathways and solution phase behavior by changing polymer solubility, amphiphilicity, and solvent selectivity. The authors further show that thin films cast from a lyotropic solution showed a significant improvement in crystallinity and crystallite size compared to those from an isotropic solution. As a result, the charge carrier mobility of those thin films can be enhanced by up to five-fold. Their subsequent work (Bridges et al., 2017) suggested that a stronger intermolecular interaction measured by the Maier–Saupe interaction parameter can result in a higher degree of macroscopic alignment as indicated by a higher dichroic ratio in thin films sheared from lyotropic solutions. A stronger intermolecular interaction was achieved by reducing the bulkiness of the side chains without losing the mesophase and rotating the side chain orientation that allows for closer π-stacking.

Kim et al. proposed a molecular design principle for promoting lyotropic LC-conjugated polymers (Kim et al., 2013). The authors suggested that three main components are required: (i) concentration-induced chain planarization, (ii) bulky side chains to prevent side-chain interdigitation and inhibit excessive backbone π–π interactions, and (iii) a tetrahedral carbon linker between the backbones and the side chains with out-of-plane bonding. The first requirement was achieved by introducing intramolecular S–F interactions that form with increasing polymer concentration. Once formed, such intramolecular interactions cause backbone planarization and consequently interchain assembly *via* π–π interaction. Besides that, bulky side chains attached to the tetrahedral carbon atom are able to prevent massive aggregation, resulting in a LC nature of polymer chains during assembly. Among four polymer analogs synthesized in this work, only the one satisfying these three design requirements possesses LC mobility and can be aligned well along the flow field. In the follow-up work (Chung et al., 2019), these three requirements were further validated. More importantly, several other structural details were examined in order to realize the direct alignment of conjugated polymers. It was found that suitable backbone planarity is required to form LC mesophase. An excessively twisted backbone conformation hampers effective molecular packing which can be required to form LC mesophase, whereas intrinsic planarity may result in massive aggregation which prevent the formation of mesophases by reducing polymer chain mobility. Besides that, the side chain branching point location is also a critical factor to prevent massive aggregation. Only a branching point near the conjugated backbone produces a sufficient steric hindrance to prevent strong intermolecular π–π interactions. On the other hand, it remains unclear whether these specific molecular design rules apply to other conjugated polymer systems. Nonetheless, it is generally recognized that hindering strong π–π interactions or side-chain interdigitation is necessary to promote lyotropic LC mesophase by preventing massive aggregation.

While recent studies have shown the important role of LC-mediated molecular assembly in the crystallization and alignment of conjugated polymers, the detailed structures of LC mesophases are rarely reported, which impedes the understanding of LC-mediated assembly pathways as there may be diverse LC mesophases. In our recent work (Park et al., 2019), we found a lyotropic, twist-bend nematic (*N*_TB_) phase from an isoindigo-bithiophene-based polymer (PII-2T) which can be readily observed through *in situ* CPOM imaging of the receding meniscus (Figure 2C). This *N*_TB_ phase was previously reported to form from twisted or bent molecules or bent-core colloids upon assembly into helical aggregates (Dozov, 2001; Mandle and Goodby, 2016). However, such *N*_TB_ phase is rarely reported in polymer systems and is not previously observed in conjugated polymers. Through comparing torsional PII-2T with a planar counterpart, we drew a link between backbone torsion and the emergence of this chiral mesophase. The twisted mesophase formed from intrinsically torsional polymer molecules is less favorable for high molecular alignment and charge transport in thin films. However, the electronic property and morphology can be significantly tuned by varying the printing flow. As shown in Figure 2C, increasing strain rates in transition regime during printing offers a possibility to stretch out the twisted backbone and thereby removes the *N*_TB_ phase. As a result, a maximum four-fold higher hole mobility is obtained compared to the films printed from the evaporation regime mediated by the *N*_TB_ phase. This is the first study to report that the LC-mediated assembly pathway can be manipulated by the printing process. This work further suggests that structural characterization and identification of LC mesophases are critical for tuning the LC phase-mediated assembly pathway in a controlled fashion.

## Conclusions and Perspectives

In conclusion, we summarize the three major molecular assembly pathways of conjugated polymers: (i) direct crystallization from dispersed polymer chains, (ii) pre-aggregation-mediated assembly pathway, and (iii) liquid crystal-mediated assembly pathway. In each case, we discuss the possible elementary steps of assembly, primary and secondary assembled structures in solution, and the resultant final thin film morphology. Valuable insights into understanding the three distinct assembly pathways and strategies to control them are provided by several studies featured in this mini-review. Specifically, lowering the solvent quality and/or temperature is a common practice to induce assembly from dispersed polymer chains. Parameters such as choice of solvent, dissolution temperature, and solution aging time offer the possibility of tuning pre-aggregation-mediated assembly pathway which critically determines the film morphology and charge transport properties. Strategies such as incorporating bulky side chains, optimizing backbone planarity, etc., have been developed to facilitate mesophase formation. Furthermore, a recent work that discovered a twist–bend nematic phase alludes to the existence of various LC mesophases of complex structures for conjugated polymers, which remain to be further explored. At the same time, many intriguing questions remain unanswered. For example, the elementary steps leading up to the emergence of the intermediate phase during assembly are rarely reported and thus remain elusive. Toward answering this question, *in situ* characterizations can provide valuable insights into the elementary steps of assembly and structural evolution of conjugated polymers during the film solidification process (Engmann et al., 2016; Ro et al., 2016; Richter et al., 2017; Persson et al., 2019). Besides these, for each type of assembly pathway, how do the precise polymer conformation and assembled structure of those intermediate phases determine solid-state morphology and properties? Moreover, what are the general molecular design principles that prescribe the near-equilibrium assembly pathways? How do various processing conditions (e.g., multiphase interfaces, fluid flow, and confined environment) alter the assembly pathways? Addressing these questions will present a significant step forward for understanding how to better control the molecular assembly of conjugated polymers and how the solid-state morphology and properties can be determined by different assembly pathways in a controllable fashion.

## Author Contributions

ZX wrote the manuscript. KP prepared Figures 1, 2 and revised the manuscript. YD provided guidance and revised the manuscript. All authors contributed to the article and approved the submitted version.

## Conflict of Interest

The authors declare that the research was conducted in the absence of any commercial or financial relationships that could be construed as a potential conflict of interest.
